# E-PSMA: the EANM standardized reporting guidelines v1.0 for PSMA-PET

**DOI:** 10.1007/s00259-021-05245-y

**Published:** 2021-02-19

**Authors:** Francesco Ceci, Daniela E. Oprea-Lager, Louise Emmett, Judit A. Adam, Jamshed Bomanji, Johannes Czernin, Matthias Eiber, Uwe Haberkorn, Michael S. Hofman, Thomas A. Hope, Rakesh Kumar, Steven P. Rowe, Sarah M. Schwarzenboeck, Stefano Fanti, Ken Herrmann

**Affiliations:** 1 Nuclear Medicine, Department of Medical Sciences, University of Turin, Turin, Italy; 2 Department of Radiology & Nuclear Medicine, Amsterdam University Medical Centers, VU University Medical Center, Cancer Center Amsterdam, De Boelelaan 1117, 1081 HV Amsterdam, The Netherlands; 3 St. Vincent’s Clinical School, University of New South Wales, Kensington, NSW Australia; 4 Department of Theranostics and Nuclear Medicine, St. Vincent’s Hospital Sydney, Darlinghurst, NSW Australia; 5 Department of Radiology & Nuclear Medicine, Amsterdam University Medical Centers, University of Amsterdam, Amsterdam, The Netherlands; 6 Department of Nuclear Medicine, University College London Hospitals NHS Foundation Trust, London, UK; 7 Ahmanson Translational Theranostics Division, Department of Molecular and Medical Pharmacology, David Geffen School of Medicine, University of California Los Angeles, Los Angeles, CA 90095 USA; 8 School of Medicine, Department of Nuclear Medicine, Technische Universität München, Munich, Germany; 9 Department of Nuclear Medicine, Heidelberg University Hospital, Heidelberg, Germany; 10 Molecular Imaging and Therapeutic Nuclear Medicine, Peter MacCallum Cancer Centre, Melbourne, VIC Australia; 11 Sir Peter MacCallum Department of Oncology, University of Melbourne, Melbourne, VIC Australia; 12 Department of Radiology and Biomedical Imaging, University of California San Francisco, San Francisco, CA USA; 13 Department of Nuclear Medicine, All India Institute of Medical Sciences, New Delhi, India; 14 Division of Nuclear Medicine, The Russell H. Morgan Department of Radiology and Radiological Science, Johns Hopkins University School of Medicine, Baltimore, MD USA; 15 Department of Nuclear Medicine, Rostock University Medical Centre, Rostock, Germany; 16 Nuclear Medicine, IRCCS Azienda Ospedaliero-Universitaria di Bologna, University of Bologna, Bologna, Italy; 17 Department of Nuclear Medicine, University of Duisburg-Essen and German Cancer Consortium (DKTK), University Hospital Essen, Essen, Germany

**Keywords:** PSMA-PET, PSMA prostate cancer, Structured report, Consensus panel, EANM guidelines, Prostate cancer guidelines

## Abstract

**Rationale:**

The development of consensus guidelines for interpretation of Prostate-Specific Membrane Antigen (PSMA)-Positron Emission Tomography (PET) is needed to provide more consistent reports in clinical practice. The standardization of PSMA-PET interpretation may also contribute to increasing the data reproducibility within clinical trials. Finally, guidelines in PSMA-PET interpretation are needed to communicate the exact location of findings to referring physicians, to support clinician therapeutic management decisions.

**Methods:**

A panel of worldwide experts in PSMA-PET was established. Panelists were selected based on their expertise and publication record in the diagnosis or treatment of PCa, in their involvement in clinical guidelines and according to their expertise in the clinical application of radiolabeled PSMA inhibitors. Panelists were actively involved in all stages of a modified, nonanonymous, Delphi consensus process.

**Results:**

According to the findings obtained by modified Delphi consensus process, panelist recommendations were implemented in a structured report for PSMA-PET.

**Conclusions:**

The E-PSMA standardized reporting guidelines, a document supported by the European Association of Nuclear Medicine (EANM), provide consensus statements among a panel of experts in PSMA-PET imaging, to develop a structured report for PSMA-PET in prostate cancer and to harmonize diagnostic interpretation criteria.

**Supplementary Information:**

The online version contains supplementary material available at 10.1007/s00259-021-05245-y.

## Introduction

Prostate-specific membrane antigen (PSMA) is one of the most successful targets for imaging and therapy in nuclear medicine. PSMA is a glycoprotein, a membrane bound metallo-peptidase, encoded by FOLH1 gene on chromosome 11. The protein acts as a glutamate carboxypeptidase on different alternative substrates, including the nutrient folate and the neuropeptide N-acetyl-l-aspartyl-l-glutamate (NAAG) and is expressed in a number of tissues such as prostate, kidney, and salivary glands [1]. The upregulation of PSMA in prostate cancer (PCa) cells is well known and is used as an effective diagnostic marker for the presence of PCa. This over-expression is present in over 90% of PCa cells, making PSMA a reliable tissue biomarker for PCa functional imaging [2]. The current hypothesis concerning the function of PSMA is that it plays a role in folate transportation and metabolism. The extra-membrane part of PSMA potentially hydrolyzes glutamated folates released by dying tumor cells. The created folate may be taken up by healthy PCa cells, facilitating further cell proliferation [1]. There is a direct effect of the PSMA receptor on the AkT and PI3K growth pathways, and it likely has a strong role as a driver of cell growth in PCa [2, 3]. PSMA expression levels increase according to the stage and tumor grade, as well as aneuploidy and biochemical recurrence (BCR). Higher levels of PSMA expression are associated with poorer prognostic outcomes [3]. More importantly, PSMA expression is upregulated when castrate-resistant phenotype evolves [4, 5].

This characteristic makes PSMA particularly attractive since it has potential as an early indicator of progression and tumor heterogeneity in castration-resistant prostate cancer (CRPC) [5]. The localization of the catalytic site of PSMA in the extracellular domain has allowed for the development of very small and highly specific inhibitors that once radiolabeled (namely with ^68^Ga or ^18^F) are used as radiopharmaceuticals for positron emission tomography (PET) imaging [6, 7]. This favorable biological and biochemical characteristic of PSMA-based PET/computed tomography (CT) imaging is a key driver among the new-generation imaging techniques [8–10].

## Clinical guidelines and previous PSMA-PET evaluation systems

Clinical guidelines in PCa have been promoted by several medical societies, namely urological, oncological, and radiation oncology societies. Recently, the European Association of Urology (EAU) guidelines on PCa [8] recommend the use of PSMA-PET imaging for any case of biochemical recurrence after radical prostatectomy (PSA > 0.2 ng/mL), namely if PSMA-PET scan is able to positively influence the subsequent treatment strategy. According to the most recent literature, BCR after primary definitive therapy represents the clinical scenario where PSMA-based imaging has the highest impact on patient management and the clinical decision-making process [11]. In this clinical setting, PSMA-PET provided superior diagnostic accuracy compared to other radiotracers, such as choline or fluciclovine [12, 13]. PSMA-based PET imaging is characterized by a high target-to-background ratio, which results in superior sensitivity and high inter-reader agreement [12, 14]. Initial staging prior to radical treatment in high-risk PCa or CRPC might be further applied for PSMA-PET, even though current clinical guidelines do not yet recommend these. Recently, both ASCO guidelines [15] and EAU consensus conference in management of advanced PCa [16] promoted the use of new-generation imaging, including PSMA-PET, for investigating advanced PCa. PSMA-PET showed high accuracy to detect PCa lesions in patients with non-metastatic PCa on conventional imaging. Finally, PSMA-PET is a key requisite in later stages of the disease considering the increasing importance of PSMA-targeted radioligand therapy (RLT) [17].

Uniform and reproducible image interpretation is important in providing comparable data between clinical trials and to meet emerging clinical diagnostic needs. While research reporting tools need to be reproducible and accurate to allow for stratification of patient cohorts or to provide the structure for pooling of data, clinical diagnostic reporting tools need to be simple and adaptable to specific clinical situations. Harmonization of PSMA-PET interpretation is also needed to communicate the exact locations of findings to referring physicians, to support clinician therapeutic management decisions, as is the case for metastasis-directed therapy.

Recently, three different criteria were published to improve objectivity and accuracy in image interpretation for PSMA-PET: EANM criteria [18], PROMISE criteria [19], and PSMA-RADS [20]. These three PSMA interpretation criteria were recently compared within an external validation [21], in order to assess the inter-reader, intra-reader, and inter-criteria agreement. The three proposed criteria have good reproducibility in evaluating [^68^Ga]Ga-PSMA-11 PET. However, there are factors leading to inter-reader disagreement indicating that further work is needed to harmonize and/or improve the interpretation criteria for PSMA-PET imaging in order to find the right balance between accuracy and the time requirements for each system.

## Radiolabeled PSMA ligands: physiological uptake, variants, diagnostic accuracy, and pitfalls

### PSMA-binding variants

[^68^Ga]Ga-PSMA-11 was introduced in 2011 by the German Cancer Research Centre [7]. As the most used radioligand, its success can be explained by the relatively straightforward isotope production (germanium-gallium generator) and radiolabeling of the tracer [22]. [^18^F]DCFPyL [23] is another widely used tracer that was introduced as a successor to [^18^F]DCFBC [24]. Compared to [^68^Ga]Ga-PSMA-11, ^18^F-labeled compounds have longer half-life, allowing imaging at later time point, a higher production capacity and a more centralized production [25, 26]. [^18^F]PSMA-1007 represents another ^18^F-tracer variant characterized by predominant hepatobiliary excretion [27], thus reducing the urinary excretion of the radiotracer. Several other PSMA ligands are available for PET imaging (e.g., [^18^F]rh-PSMA7, [^68^Ga]Ga-PSMA I&T, [^18^F]JK-PSMA-7, [^68^Ga]Ga-PSMA-R2), but few data in literature are available at present [28, 29].

### Physiological uptake

For [^68^Ga]Ga-PSMA-11 and [^18^F]DCFPyL, high PSMA uptake is noted in the cortex of the kidneys, parotid and submandibular salivary glands, and duodenum. Moderate median uptake (SUV_max_ >3) is noted in the spleen, liver, and lacrimal glands [30]. In comparison to [^68^Ga]Ga-PSMA-11, the uptake of [^18^F]PSMA-1007 is higher in the liver, gallbladder, pancreas, and sublingual glands, and lower in the kidneys, bladder, and lacrimal glands [31]. Since daily repeatability on the PSMA-ligand uptake for each organ is essential for semi-quantitative analysis, knowledge on dosimetry and physiological uptake is important [32]. Moreover, knowledge about uptake in benign tissues is important, by enabling to calculate the thresholds that can classify potential malignant lesions when interpreting PET-scans [32]. Kidney, spleen, and salivary uptake are higher on [^68^Ga]Ga-PSMA-11 compared to [^18^F]DCFPyL, while the liver shows slightly lower uptake. The blood pool (aorta) is similar in both [^18^F]DCFPyL and [^68^Ga]Ga-PSMA-11 and can therefore be used as a benchmark in assessing lesions based on SUV_max_ [32]. One limitation of both [^68^Ga]Ga-PSMA-11 and [^18^F]DCFPyL is their increased urinary excretion in the ureters and the bladder, which limits detection of local recurrence in the prostate bed. This excretion is absent for [^18^F]PSMA-1007 due to hepatobiliary clearance, and might improve local detection of PCa [31].

### Pitfalls

The expression of PSMA can predominantly be found in PCa, but benign and other malignant tissues are known to express PSMA and have extensively been described [33]. Here we present an array of pitfalls that can influence reader’s interpretations. Although the positive predictive value and specificity of PSMA-PET are known to be high [14, 34], cautious reading and knowledge of common pitfalls should be considered while interpreting PSMA-PET images and drafting the medical report.

#### Other malignancies

While epithelial PSMA expression is the mechanism for detecting PCa, for other neoplasms, it is hypothesized to be associated with tumor neovasculature [35]. Renal cell carcinoma (RCC) is most commonly described, especially in clear cell RCC [36]. Although it may present as a pitfall, it also creates the opportunity to utilize PSMA-PET in the detection of these malignancies, including RCC [36], hepatocellular carcinoma [37], breast cancer, and lung cancer [38] and other malignancies. These indications are strictly restricted to dedicated research protocols and do not have clinical use yet.

#### Ganglia

Ganglia might be considered one of the most common pitfalls, since they can mimic lymph nodes. In both [^18^F]DCFPyL and [^68^Ga]Ga-PSMA-11, PSMA expression was observed in at least one ganglion (96.9–98.5% of all scans) [39]. Since ganglia can mimic the anatomical locations of lymph nodes, knowledge of their presence is important for accurate staging, thus preventing false interpretation. Ganglia should be distinguished from lymph node metastases based on location, tracer uptake, and configuration. Most important for their differential diagnosis, ganglia almost never have a nodular appearance (2.0%) such as metastatic lymph nodes (58.6%), and are usually linear shaped (71.2%) [39].

#### Benign bone disease

Most common pitfalls when differentiating bone metastases (M1b) from other pathologies are healing bone fractures and degenerative bone changes [40, 41]. [^18^F]PSMA-1007 has been described as expressing increased PSMA expression not only in benign bone lesions compared to [^68^Ga]Ga-PSMA-11, specifically fractures, but also in non-trauma-related PSMA uptake [41]. Several case reports have been published for [^18^F]DCFPyL and [^68^Ga]Ga-PSMA-11, showing Paget’s disease localizations in the pelvis, humerus, and sacrum [42]. Other reasons reported in the literature are fibro-osseous lesions (e.g., fibrous dysplasia and hemangiomas), which are most commonly present in the ribs [43].

#### Benign neurogenic tumors

Brain parenchyma normally does not contribute to PSMA uptake, therefore giving the clinician excellent visualization of potential PSMA expressing metastases. However, there have been several case reports of pitfalls in [^68^Ga]Ga-PSMA-11 scans, with PSMA expression seen after ischemic strokes [44]. In addition, gliomas, meningiomas, paragangliomas, and neurofibromas have been described to have PSMA uptake [45, 46]. Focal uptake in the brain parenchyma on PSMA-PET generally requires MRI correlation.

#### Pulmonary sarcoidosis/granulomatosis

Although the uptake mechanisms are not understood, several case reports have described PSMA-ligand uptake in pulmonary sarcoidosis [47]. Another chronic granulomatosis inflammatory disorder that is known to express PSMA is Wegener’s granulomatosis [48]. Other inflammatory lung conditions that are known to express PSMA in [^68^Ga]Ga-PSMA-11 scans are bronchiectasis, anthracosilicosis, and tuberculosis [49–51].

#### Androgen activity

Androgen receptor (AR) inhibition is believed to increase PSMA expression in PCa [4, 5]. This upregulation and its exact timing are not completely understood but must be considered to prevent falsely defining disease progression shortly after initiation of AR-targeted therapies. This increase in PSMA uptake appears transient, since it is more visible during the first weeks of hormonal blockade, with a tendency to decrease over time [5, 52]. However, PSMA upregulation may not be transient, but PSMA-ligand uptake goes down due to treatment response rather than downregulation of PSMA ligand.

Gynecomastia shows increased PSMA uptake and might be observed in patients undergoing ADT [53].

#### Decreased PSMA expression

Although most pitfalls represent potential false-positive findings, some factors may induce potential false-negative PSMA findings, as well. Up to 5% of prostate adenocarcinomas do not express PSMA. Furthermore, aggressive forms of primary neuroendocrine PCa or neuroendocrine de-differentiation after AR-targeted therapies in metastatic CRPC (mCRPC) might show a reduced PSMA expression in metastatic sites [54].

## Methodology: the consensus panel

### Rationale of the study

The development of consensus guidelines for interpretation of PSMA-PET may contribute to provide more consistent reports in clinical practice. The standardization of PSMA-PET interpretation may also contribute to increasing the data reproducibility within clinical trials. Defined criteria for interpreting PSMA-PET images would help improving accuracy, precision, and repeatability of this diagnostic procedure, thus improving patients’ management and outcomes. Therefore, consensus interpretation is necessary to provide comparison between clinical trials and to meet upcoming clinical diagnostic needs. Consensus guidelines in PSMA-PET interpretation are also needed to communicate the exact location of findings to referring physicians, to support clinician therapeutic management decisions, as happens for MDT.

In view of these considerations, the E-PSMA standardized reporting guidelines, a document supported by the European Association of Nuclear Medicine (EANM), is aimed at providing consensus statements among a panel of experts in PSMA-PET imaging, to develop a structured report for PSMA-PET in PCa and to harmonize diagnostic interpretation criteria.

### Panel composition

According to the abovementioned purposes, a panel of worldwide experts in PSMA-PET was established. Panelists were selected based on their expertise and publication record in the diagnosis or treatment of PCa, in their involvement in clinical guidelines and according to their expertise in the clinical application of radiolabeled PSMA inhibitors. The panelists involved are reported in Table 1.

**Table 1:** Panel composition

Name	Role	Institution	Country
Judit Adam	EANM Oncology and Theranostics committee representative	Amsterdam UMC, University of Amsterdam	The Netherlands
Jamshed Bomanji	Panelist	University of London	UK
Francesco Ceci	Project coordinator	University of Turin	Italy
Johannes Czernin	Panelist	University of California Los Angeles	USA
Matthias Eiber	Panelist	Technical University of Munich	Germany
Louise Emmett	Panelist	St. Vincent Hospital, Sydney	Australia
Stefano Fanti	Project leader	University of Bologna	Italy
Uwe Haberkorn	Panelist	University of Heidelberg	Germany
Ken Herrmann	Project leader	University Hospital of Essen	Germany
Michael Hofman	Panelist	Peter MacCallum Cancer Centre, Melbourne	Australia
Thomas Hope	Panelist	University of California San Francisco	USA
Rakesh Kumar	Panelist	All India Institute Of Medical Sciences, New Delhi	India
Daniela Oprea-Lager	Panelist	Amsterdam UMC, Vrije Universiteit Amsterdam	The Netherlands
Steven Rowe	Panelist	Johns Hopkins Baltimore	USA
Sarah Schwarzenboeck	Panelist	University of Rostock	Germany

### Modified Delphi consensus process

**Fig. 1:**
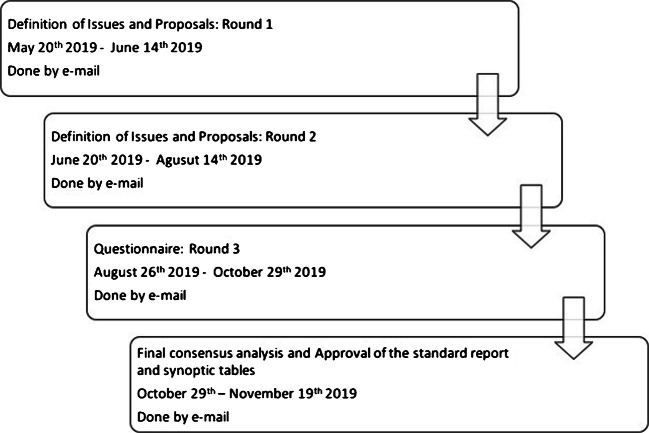
E-PSMA project timeline

**Fig. 2:**
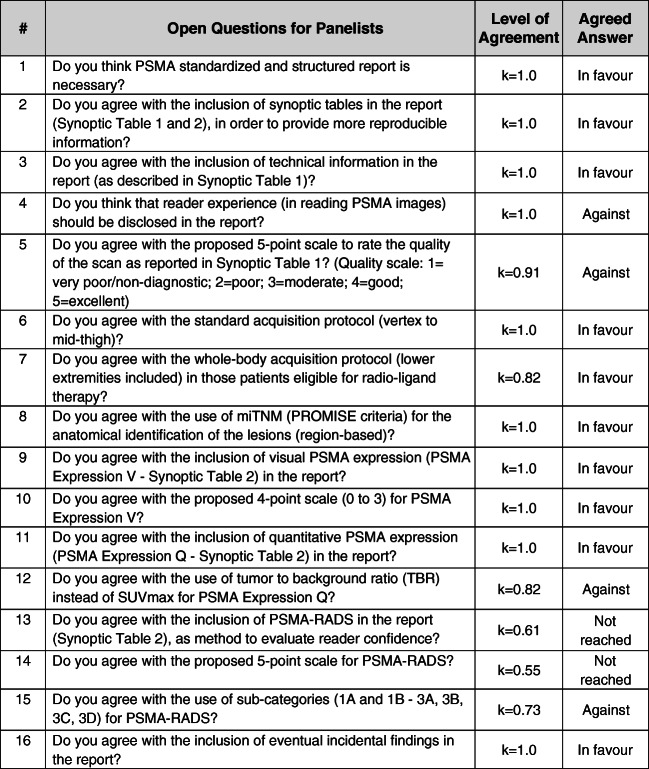
Open questions for panelists

Panelists were actively involved in all stages of a modified, non-anonymous, Delphi consensus process, as displayed in Fig. 1. In the first two rounds, panelists identified issues regarding PSMA-PET reporting and made proposals about possible criteria to harmonize and standardize the PSMA-PET reporting process. Finally, panelists gave their inputs to identify which criteria or parameters currently used in research studies might be implemented in clinical reports. According to comments, proposal, and suggestions made by panelists during the first and second round, a questionnaire (Fig. 2) composed by 16 questions has been generated. All panelists were asked to answer the questions as in favor or disagree. The inter-rater agreement was measured for each question using Fleiss’ kappa (0 poor agreement; 0.01–0.20 slight agreement; 0.21–0.40 fair agreement; 0.41–0.60 moderate agreement; 0.61–0.80 substantial agreement; 0.81–1.00 almost perfect agreement).

Fourteen out of sixteen questions reached an almost perfect concordance between panelists. Question nos. 13 (*k* = 0.61) and 14 (*k* = 0.55) reached only a moderate agreement between experts. At question nos. 1, 2, 3, 6, 7, 8, 9, 10, 11, and 16, the agreement was in favor of the topic proposed. Therefore, structured report, synoptic tables, technical information, type of acquisition, staging criteria, visual and quantitative PSMA expression, and incidental findings have been included in E-PSMA reporting guidelines.

At questions 4, 5, 12, and 15, the agreement was against the topic proposed. Therefore, reader experience, quality of the scan, TBR instead of SUV_max_, and the use of sub-categories in the 5-point scales were not included in E-PSMA reporting guidelines. Since questions 13 and 14 did not reach consensus, PSMA-RADS criteria [20] were not included in the final report. However, considering comments and proposal made in rounds 1 and 2, all panelists agreed in the inclusion of a modified 5-point scale to reflect the likelihood of the presence of PCa-related lesions.

## The EANM standardized reporting guidelines: E-PSMA—imaging methodology, structured report, and synoptic tables

According to the findings obtained by modified Delphi consensus process, panelist recommendations were implemented in a structured report for PSMA-PET. Expert recommendations are reported below. PSMA-PET report template is reported in Appendix 1 (Supplemental Material). The synoptic tables are reported in Appendix 2 and 3 (Supplemental Material).

### Imaging methodology

Many technical factors relating to methodology may affect the quality of a PET image acquisition. As these methodological aspects may influence the quality and interpretation of PSMA-PET images, they should be described in each PSMA-PET report. The head of the report should include synoptic tables to summarize PSMA-PET technical data (synoptic Table 1).

Tracer activity used should be reported in MBq, whether fixed (333 MBq for [^18^F]DCFPyL) or patient-specific (1.8–2.2 MBq/Kg for [^68^Ga]Ga-PSMA-11 and 4 MBq/Kg for [^18^F]PSMA-1007). In case a different tracer dosage is used, this should be reported.

As tumor PSMA uptake does not plateau within commonly used intervals, uptake intervals may affect lesion-to-background contrast, detection rates, and quantitative reads. Therefore, the uptake interval between tracer injection and imaging should be reported. When used, type of diuretics and dosage should be reported.

The type of CT protocol (low dose vs. diagnostic) and use of oral or intravenous contrast should be reported. If intravenous contrast is used, consideration should be given to imaging in the urography phase to better delineate the ureters and bladder, in the staging or biochemical recurrence setting.

The standard acquisition should be from the vertex to the mid-thigh. In patients where there is known disease or concern for disease in the lower extremities, the acquisition can be extended (total-body acquisition). Total-body acquisition can be considered also in patients eligible for radioligand PSMA-based therapy.

The overall quality of the study should be assessed. This judgment is based on reader personal experience and it is aimed to evaluate the reproducibility of the scan. However, in consideration of experts’ recommendation, this information should not be included in the report.

### The structured report

An overview of all items necessary for reporting of findings on PSMA-PET that can be clinically used is provided in synoptic Table 2. Synoptic Table 2 should be included at the end of the clinical report as the final summary.

**Table 2:** Qualitative evaluation of PSMA expression through a 4-point scale

PSMA expression V (visual score)	Grade of PSMA expression
Score = 0	Below blood pool
Score = 1	Equal to or above blood pool and lower than liver
Score = 2	Equal to or above liver and lower than parotid gland
Score = 3	Equal to or above parotid gland

#### Patient history

Depending on the clinical indication for PSMA-PET, several variables in a patient’s history will influence his a priori chance of having primary prostate cancer, local recurrence, lymph node metastases, or distant metastatic disease [55–58].

In newly diagnosed patients, clinical tumor stage, PSA level, and Gleason score influence the probability of having lymph node metastases [59, 60]. Also at biochemical recurrence, the probability of scan positivity will depend on the patients’ PSA level and its kinetics (namely PSA doubling time (PSAdt)), together with the clinical setting of PSA relapse (biochemical persistence vs. first biochemical recurrence vs. advanced metastatic disease) [55–58, 61, 62]. Therefore, knowledge of risk factors such as PSA level, PSAdt, Gleason score, clinical or pathological tumor stage, and prior and/or current treatment(s) is necessary to adequately report on findings on PSMA-PET.

ADT might modulate levels of PSMA expression over time, and its use and timing should be included in reports, especially for comparison of longitudinally repeated PSMA-PET [5, 52].

The clinical setting of recurrence (biochemical persistence vs. biochemical recurrence vs. advanced disease) as well as the scenario of hormone-sensitive PCa (HSPC) vs. CRPC should be included as well [55, 57].

#### General consideration

Describing PSMA uptake in either prostate, prostate bed, or metastases (lymph node, bone, or visceral soft tissue) should include both qualitative and quantitative descriptions, as reported in synoptic Table 2. Visual description (PSMA expression V) should relate PSMA uptake to background uptake in the blood, liver, and salivary glands on a visual scale 0–3, as reported in Table 2. Quantitative description (PSMA Expression Q) should preferably include SUV_max_ or, alternatively, a tumor-to-background ratio.

According to experts’ recommendation (question 8), reports on primary tumor/prostate bed recurrence and metastases should include TNM classification (molecular imaging TM (miTNM)), as proposed by PROMISE criteria [19] and as reported in Table 3.

**Table 3:** Regional classification of PSMA-PET findings

Class	Description
Local tumor (T)	
miT0	No local tumor
miT2	Organ-confined tumor
miT3a	Non-organ-confined tumor (extracapsular extension)
miT3b	Non-organ-confined tumor (seminal vesicles invasion)
miT4	Tumor invading adjacent structures (other than seminal vesicles)
miTr	Presence of local recurrence after radical prostatectomy
Regional nodes (N)	
miN0	No positive regional lymph nodes
miN1	Positive regional lymph nodes
Distant metastases (M)	
miM0	No distant metastases
miM1a	Extra-pelvic lymph nodes
miM1b	Bone metastasis
miM1c	Non-nodal visceral metastasis: report involved organ(s)

Adapted from Eiber M, et al. Prostate cancer molecular imaging standardized evaluation (PROMISE): proposed miTNM classification for the interpretation of PSMA-ligand PET/CT. J Nucl Med. 2018 Mar;59(3):469–478

Suspected PCa site(s) (e.g., prostate/prostate bed, lymph node station, bone structure, organs), anatomical size, and number of lesions (oligo vs. multi-metastatic disease) should be reported.

Finally, the report should include a 5-point scale [20], as framework for classifying individual findings into categories that reflect the likelihood of the presence of PCa, as reported in Table 4. The number of findings should be reported for each region, as defined by the miTNM criteria. In case of multiple metastases (e.g., more than five), as might happen in advanced patients, readers can add the definition of “multi-metastatic or poly-metastatic” in the specific region of dissemination. In case of poly-metastatic disease, data about lesion quantification should be calculated considering the five most evident metastases per region (to be selected based on size and intensity).

**Table 4:** Interpretation of PSMA-PET findings according to the reader confidence expressed through a 5-point scale

Score	Definition
1	Benign lesion without abnormal PSMA uptake
2	Probably benign lesion: faint PSMA uptake (equal or lower than background) in a site atypical for prostate cancer
3	Equivocal finding: faint uptake in a site typical for prostate cancer or intense uptake in a site atypical for prostate cancer
4	Probably prostate cancer: intense uptake in typical site of prostate cancer, but without definitive findings on CT*
5	Definitive evidence of prostate cancer: intense uptake in typical site of prostate cancer, with definitive findings on CT

Adapted from Werner RA, et al. Recent updates on molecular imaging reporting and data systems (MI-RADS) for theranostic radiotracers-navigating pitfalls of SSTR- and PSMA-targeted PET/CT. J Clin Med. 2019 Jul 19;8(7)

*A definitive finding on CT means the presence of a real anatomical substrate on the CT

#### Region-based analysis: prostate and prostate bed

In the initial stage of PCa, reporting on local tumor stage should include uni vs. multifocal disease, laterality (unilateral or bilateral), localization (apical, median, or basal), and (whenever possible) suspicion of extracapsular extension (ECE) or seminal vesical invasion (SVI). Reporting ECE and SVI should preferably include anatomical description from diagnostic CT (if available). Reporting on potential recurrence in prostate bed should include laterality and localization (anastomosis vs. posterior) and uni vs. multifocal disease.

#### Region-based analysis: lymph nodes

Lymph node size is not always directly related to chance of PCa metastases [63]. Still, the likelihood of PET positivity does depend on size, as microscopic metastases (e.g., ⩽3 mm) will likely remain below PET detection limits. This contributes to false-negative findings of PSMA-PET for lymph node staging in primary PCa [59, 60, 64]. In the biochemical recurrence setting, as histopathological confirmation is often lacking, caution for false positives is mandated [33, 39]. Reporting should include the exact station of lymph node metastases and the short-axis measurement. Also, pelvic lymph node descriptions should include where nodes are located, to help urologist to interpret whether PSMA-PET-positive lymph nodes are within or outside surgical dissection templates. Whenever possible, chance of malignancy should be related to quantitative measurements and reader’s confidence (Table 4).

#### Region-based analysis: skeleton

One of the pitfalls of PSMA-PET is the rate of false-positive bone lesions. The exact cause of false positivity is not known, but may be attributed to bone degeneration, traumatic injury, or benign bone lesions. It should be noted that false positivity rate for bone lesions may differ between PSMA ligands [41]. Solitary bone lesions detected in patients with no metastases otherwise should be interpreted with caution, especially in pre-surgery setting. Reports should include clinical characteristics (e.g., pain, PSA kinetics), and correlation with other modalities including MRI, contrast-enhanced CT, and bone scan. When there is uncertainty and histopathological confirmation is feasible, it should be recommended to referring physicians in reports.

#### Region-based analysis: non-nodal visceral soft tissue

Detection or suspicion of visceral metastases should include reporting of localization and PSMA expression levels in relation to background uptake qualitatively (PSMA expression V, Table 2) and quantitatively (PSMA expression Q). Presence of visceral metastases should be reported with anatomical information from CT and related to clinical characteristics (e.g., PSA levels and comorbidities), to prevent detection of false positives [33].

## E-PSMA reporting system in staging, recurrent setting, advanced setting, and response to therapy: a clinical summary

The use of PSMA-PET is increasing in routine clinical practice both in initial staging of PCa and for the localization of biochemically recurrent PCa. The accurate detection of malignant PCa lesions has a major impact on management decisions and may result in withholding definitive local therapy or lead to metastasis-directed therapy [65].

### Primary staging

In primary staging, early detection of metastases is essential. Patients with proven metastatic disease are usually treated differently than patients with localized PCa. The detection of any additional lesion may change patient management and result in local radiotherapy, extended lymph node dissection, (oligo)metastases-directed therapy, or systemic (palliative) treatment. In a systematic review [66], high variation in sensitivity (33–92% on a per-lesion analysis) with overall optimal specificity (82–100% on a per-lesion analysis) was found in the detection of lymph node metastases, correlated by histopathological evaluation (extended pelvic lymph node dissection). Additionally, the primary tumor is nearly always detected by PSMA-PET, and PET metrics correlated with histologic grades (ISUP classification) [67, 68]. Analyses regarding the diagnostic accuracy of ^18^F-labeled PSMA-PET in primary staging are ongoing. In yet unpublished results of a prospective trial, a sensitivity of 30.6–41.9% of [^18^F]DCFPyL PET for the detection of lymph node metastases was determined [68]. Regarding [^18^F]1007-PSMA-PET, local staging appears a promising technique, considering the low urinary excretion of this radiotracer [69].

Recently, in an Australian, multi-center, randomized, phase III clinical trial (proPSMA) [70], [^68^Ga]Ga-PSMA-11 PET provided greater accuracy in identifying nodal and distant metastases vs. conventional imaging (CT and bone scan) prior to curative-intent surgery or radiotherapy in high-risk PCa. Furthermore, [^68^Ga]Ga-PSMA-11 PET vs. conventional imaging was associated with change in management in 28% vs. 15% of patients and was associated with a lower percentage of equivocal findings (7% vs. 23%). Finally, even if both imaging techniques involve exposure to radiation, the dose associated with [^68^Ga]Ga-PSMA-11 was less than half that associated with conventional imaging (8.4 mSv vs. 19.2 mSv).

Regarding these considerations, PSMA-PET is a suitable replacement for conventional imaging, providing superior accuracy, to the combined findings of CT and bone scanning, in patients with high risk of nodal involvement [71], while patients at lower risk should be spared by this imaging procedure.

### Recurrent setting

The detection rate of metastases (i.e., percentage positive scans) of PSMA-PET in patients with BCR has been studied intensively. A recent meta-analysis [11] showed an overall detection rate in patients with BCR of 76%. At low PSA values (<0.5 ng/mL), detection of metastases was 45%. Furthermore, a recently published large prospective study showed comparable results [14]. The positive predictive value (PPV) of [^68^Ga]Ga-PSMA-11 PET has been calculated as 92% [14]. For ^18^F-labeled PSMA (namely [^18^F]DCFPyL and [^18^F]PSMA-1007), fewer results concerning the diagnostic accuracy in patients with BCR are available at present. For [^18^F]DCFPyL, detection rates ranging from 84.6 to 86.3% have been documented, with a detection at low PSA values (<0.5 ng/mL) of 60% [62, 72]. Preliminary results of a large prospective multicenter trial [73] showed a PPV of 84.5% for [^18^F]DCFPyL PET. Similar results have been described for [^18^F]PSMA-1007 [74, 75].

At present, EAU guidelines suggest performing PSMA-PET in any case of proven BCR [8]. However, the incidence of false-negative scans is not negligible. In recurrent setting, PSMA-PET detection rate is influenced by several factors as recently reported in some prediction models [55, 56, 58, 76]. PSA, as expression of tumor burden, is not the only influencing parameter. PSAdt and Gleason score as expression of tumor aggressiveness together with the administration of concurrent ADT are parameters able to influence the likelihood of a positive scan [76]. While reporting PSMA-PET in recurrent setting, also the clinical stage of the disease should be taken into consideration [55, 57]. Persistent disease after surgery (detectable PSA levels after surgery) [61] and BCR (undetectable PSA levels after surgery), while both represent an early recurrence, are two conditions with different outcome and different incidence of detectable metastatic disease [55, 57, 61]. Finally, the proper knowledge of potential pitfalls during PET image interpretation, while probably reducing PSMA-PET sensitivity, will increase its overall specificity [33].

### Advanced setting and assessment of the response to systematic therapy

The role of PSMA-PET in advanced setting presents less level of evidence compared to initial staging and BCR. Non-metastatic CRPC (nmCRPC) is a condition characterized by a rising PSA level, castrate testosterone levels, and no evidence of distant metastases by conventional bone scan and cross-sectional imaging of the chest, abdomen, and pelvis [8]. This clinical scenario became recently of high interest, since new androgen receptor-targeted therapies have been recently approved in this stage (SPARTAN, PROSPER, and ARAMIS trials). In the setting, PSMA-PET proved its ability to detect PCa locations in patients negative at conventional imaging (nmCRPC). Thus, as recently stated by the EAU consensus panel in advanced PCa [16], ASCO guidelines [15], and Advanced PCa Consensus Conference (APCC) [9], it should also be recognized that the majority of patients in clinical trials who benefited from the addition of next-generation ADT would probably have had positive PSMA-PET imaging results. It is uncertain whether stratification based on PSMA-PET would identify subgroups of patients (e.g., those with distant rather than local or loco-regional disease) that benefit most. Tumor heterogeneity is a key event in advanced PCa. Tumor cells exhibit different phenotypes and, accordingly, PSMA might not be over-expressed in all metastatic sites. This event should be taken into consideration while reporting PET scan in mCRPC, namely while evaluating the response to systemic therapy.

Regarding the response to therapy assessment, the Prostate Cancer Clinical Trials Working Group (PCWG) criteria include clinical and laboratory parameters, as well as conventional imaging modalities such as CT and bone scan findings but advanced molecular imaging techniques are not yet considered. PSMA-PET is not yet validated for response assessment, especially in the context of clinical trials [77]. Recently, consensus statement criteria for response evaluation using PSMA-PET were developed [16]. The statements regarded both the utility and the best time to perform PSMA-PET, as well as the optimal strategy to select patients who may benefit from treatments and criteria to be used for evaluation of response when using different types of PSMA tracers. Consensus was met on the utility of PSMA-PET for response assessment in patients with metastatic PCa, irrespective of the moment and type of treatment used (i.e., local or systemic), but solely in cases when clinical management is expected and with a 3-month interval after initiation of therapy in HSPC. Proposed criteria should only be adopted in the context of clinical trials, preferably by dividing patients in responders and non-responders. The category of responders is including the whole spectrum of patients presenting with stable disease, partial and complete response, while the non-responders are patients with progressive disease on PSMA-PET imaging. The pre-requisite of robust and reproducible interpretation of response to treatment when using PSMA-PET scans is adequate semi-quantitative evaluation. Consensus was reached on the use of SUV parameters for this purpose, by optimizing and harmonizing the protocols. Tools to estimate the total tumor burden represent a feasible alternative to reduce interobserver variability, being currently developed. However, one major issue is how to best define disease progression. Recently, PSMA-PET Progression (PPP) criteria have been proposed [78]. PPP defines PSMA treatment response in three different criteria: (1) appearance of 2 or more new PSMA-positive distant lesions, (2) appearance of 1 new PSMA-positive lesion plus consistent clinical and/or laboratory data and recommended confirmation by biopsy or correlative imaging within 3 months of PSMA-PET, and (3) increase in size or PSMA uptake of 1 or more existing lesions by 30% plus consistent clinical and/or laboratory data and/or confirmation by biopsy or correlative imaging within 3 months of PSMA-PET. These criteria should be taken into consideration while reporting PSMA-PET in patients undergoing systemic therapies.

Finally, the growing interest in PSMA-targeted therapies is not unnoticed. One phase 3 trial (VISION, NCT03511664) and one phase 2 trial (TheraP, NCT03392428) [79] are currently ongoing. Both trials use PSMA-PET to identify patients with high PSMA expression, who are suitable candidates for [^177^Lu]Lu-PSMA-617 therapy, but they use different PET imaging thresholds to define suitability. In addition, the TheraP trial uses 2-[^18^F]FDG PET to assist in identifying sites of PSMA-negative disease that cannot be targeted with [^177^Lu]Lu-PSMA-617. These patients have been shown to have a poor prognosis [80]. Finally, studies indicate that both intrapatient and interpatient PSMA expressions are highly heterogeneous in patients candidate for PSMA-targeted therapy, and that many of them express little or no PSMA. This consideration should be clearly stated and defined while interpreting PSMA-PET in advanced setting [81, 82].

## Supplementary information

ESM 1 (DOCX 649 kb)
