# Supplementary material for: E-PSMA: the EANM standardized reporting guidelines v1.0 for PSMA-PET
Source: Eur J Nucl Med Mol Imaging. 2021 Feb 19;48(5):1626–38. doi: 10.1007/s00259-021-05245-y (PMC8113168; doi:10.1007/s00259-021-05245-y)
Supplement: Supplementary file 1 — (DOCX 649 kb) [file 259_2021_5245_MOESM1_ESM.docx]

**E-PSMA: The EANM standardized reporting guidelines v1.0 for PSMA PET**

**Supplemental Materials**

**Index**

1. Page 2: Appendix 1 - PSMA PET/CT Report Template.
2. Page 4: Appendix 2 – Synoptic Tables – PET scan Technical Parameters according to different PSMA radiopharmaceuticals
3. Page 5: Appendix 3 – Synoptic Tables – Reporting on PSMA PET/CT Findings
4. Page 6: Examples of PSMA PET/CT reports according to E-PSMA
5. Page 10: Synoptic Tables Template
6. **Appendix 1 - PSMA PET/CT Report Template**

**Radiolabeled-PSMA PET/CT Study**

1. Patient History

PET/CT indication: Staging (prior to primary therapy), Restaging (biochemical recurrence or biochemical persistence), Restaging (advanced setting, CRPC), Therapy Monitoring

Clinical info: Gleason, TNM, PSA at diagnosis, Previous Primary/Salvage Therapy, PSA nadir after therapy, recent PSA values, PSAdt, Clinical Stage, Concurrent/Previous Systemic Therapy.

2. Technical Information

The patient was given *X* MBq/mCi of Radiolabeled-PSMA (change to type of radiotracer used) and imaging of the body from the vertex of the skull to the mid-thigh was obtained after *X* minutes with concurrent diagnostic/low-dose CT for attenuation correction and anatomical localization. Oral/intravenous contrast was administered/was not administered; state phase of intravenous contrast if administered.

Furosemide (*X* mg) i.v. was/was not administered.

3. Reporting of findings

Prostate/Prostate bed: This part of the report should give enough information to enable a ‘PET T stage’ Tx – T4 (T1 not applicable).

It should also identify unifocal or multifocal disease.

An intensity score of the primary lesion is likely to be an independent predictor of patient outcomes and incorporated into future nomograms, so an intensity score (either TBR or SUVmax) should be detailed.

Extraprostatic extension: yes/no, seminal vesicle(s) involvement yes/no, adjacent tissue involvement yes/no (localization).

Lymph nodes: There are no PSMA avid, enlarged or abnormal nodes in the pelvis (obturator, presacral, mesorectal, perivesical, internal/external iliac), common iliac, retroperitoneum, retrocrural space or elsewhere above the diaphragm.

**Or**

PSMA positive lymph nodes are identified as detailed below:

**
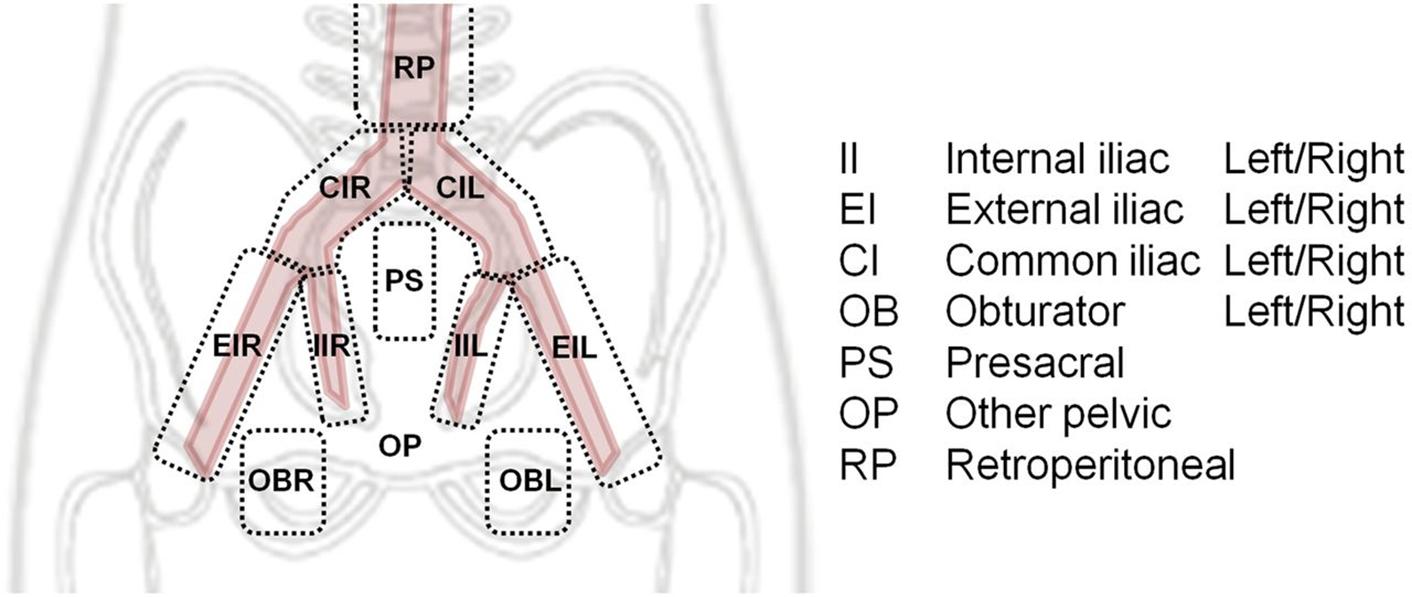
**

*(miTNM standard template for pelvic lymph node regions. Eiber M. et al. J Nucl Med. 2018 Mar;59(3):469-478)*

Osseous Disease: No focal positive PSMA lesions identified in the axial or appendicular skeleton:

**Or**

PSMA positive lesions in sclerotic or lytic lesions or bone marrow (no morphological changes in CT) identified as detailed below.

Non-Osseous Disease:

1. Identification of visceral involvement
2. Incidental findings

Conclusion:

1. Final recommendation
2. Eventual other findings (not related to prostate cancer).
3. **Appendix 2 – Synoptic Tables – PET scan Technical Parameters according to different PSMA radiopharmaceuticals**

**1a) [^68^Ga]Ga-PSMA-11**

| Radiotracer | Activity Injected | Uptake  time | Type of acquisition | CT protocol | Oral / IV Contrast | Diuretic |
| --- | --- | --- | --- | --- | --- | --- |
| [^68^Ga]Ga-PSMA-11 | 1.8–2.2 MBq/Kg | 60 – 90 min | Standard or whole-body | Diagnostic/  Non-Diagnostic | Yes/ No | Yes/ No  (10mg/ 20 mg) |

**1b) [^18^F]DCFPyL**

| Radiotracer | Activity Injected | Uptake  time | Type of acquisition | CT protocol | Oral / IV Contrast | Diuretic |
| --- | --- | --- | --- | --- | --- | --- |
| [^18^F]DCFPyL | 333±37 MBq | 60-120 min | Standard or whole-body | Diagnostic/  Non-Diagnostic | Yes/ No | Yes/ No  (10mg/  20 mg) |

**1c) [^18^F]PSMA-1007**

| Radiotracer | Activity Injected | Uptake time | Type of acquisition | CT protocol | Oral / IV Contrast | Diuretic |
| --- | --- | --- | --- | --- | --- | --- |
| [^18^F]PSMA-1007 | 4MBq/Kg | 90-120 min | Standard or whole-body | Diagnostic/  Non-Diagnostic | Yes/ No | Yes/ No  (10mg/  20 mg) |

1. **Appendix 3 – Synoptic Tables – Reporting on PSMA PET/CT Findings**

| **Anatomical location** | **mi TNM**  **^[a]^** | **Size** | **Number** | **PSMA Expression Q (*SUVmax*)** | **PSMA**  **Expression V** ^[^**^b]^** | **Reader Confidence ^[c]^** |
| --- | --- | --- | --- | --- | --- | --- |
| Prostate/  Prostate bed |  |  |  |  |  |  |
| **Anatomical location** | **mi TNM** | **Size** | **Number** | **PSMA Expression Q** | **PSMA**  **Expression V** | **Reader Confidence** |
| Region 1 |  |  |  |  |  |  |
| Region 2 |  |  |  |  |  |  |
| **Anatomical location** | **mi TNM** | **Size** | **Number** | **PSMA Expression Q** | **PSMA**  **Expression V** | **Reader Confidence** |
| Region 1 |  |  |  |  |  |  |
| Region 2 |  |  |  |  |  |  |
| **Anatomical location** | **mi TNM** | **Size** | **Number** | **PSMA Expression Q** | **PSMA**  **Expression V** | **Reader Confidence** |
| Region 1 |  |  |  |  |  |  |
| Region 2 |  |  |  |  |  |  |
| **Incidental Findings** | **Site** | **Number** | **PSMA Expression Q** | **PSMA Expression V** | **Interpretation** | **Other malignancy** |
| Yes/No |  |  |  |  |  | Suspected: Yes/No |

[a]: miTNM Classification. Table 3.

[b]: Visual Score for PSMA Expression. Table 2.

[c]: 5-point scale for reader confidence. Table 4.

1. **Examples of PSMA PET/CT reports according to E-PSMA**

Patient-1 (Name, Surname, Age, Date of birth)

**Patient History**

Prostate Cancer, Gleason Score 4+3, ISUP 3, cT3NxMx, initial PSA level 113 ng/mL. mp-MRI performed 5/2020: one lesion PIRADS-5 in the right prostate lobe. One suspected external iliac lymph node (8mm). Anamnestic: low back pain. Diabetes mellitus II.

PSMA PET indication: staging prior to radical prostatectomy.

**Technical information**

The patient was given 330 MBq [^18^F]DCFPyL intravenously. Imaging of the body from the vertex to mid-thigh was obtained after 120 minutes with concurrent diagnostic CT for attenuation correction and anatomical correlation.

| **Radiotracer** | **Activity Injected** | **Uptake**  **time** | **Type of Acquisition** | **CT Protocol** | **Contrast Oral/ IV** | **Diuretic** |
| --- | --- | --- | --- | --- | --- | --- |
| **[^18^F]DCFPyL** | 330 MBq | 120 min | Standard | Diagnostic | No | No |

**Reporting of Findings**

This report has been produced in according to the European Association of Nuclear Medicine (EANM) standardized reporting guidelines v1.0 for PSMA PET/CT.

The physiological biodistribution of the radiotracer was regular.

- Prostate: diffuse, intense PSMA uptake in the peripheral, dorsal zone of the right lobe (median and apical). PSMA intense uptake in the right seminal vesical, without any substrate on the diagnostic CT. No suspicion of left lobe and left seminal vesical invasion or extracapsular extension. No involvement of adjacent tissue.

- Lymph nodes: focal PSMA uptake in one right external iliac lymph node (8 mm). Just adjacent, there is another right external iliac lymph node (5 mm) with focal increased PSMA expression. No other suspected lymph nodes have been observed.

- Osseous disease: There is focal, increased PSMA expression in a sclerotic lesion ventral in the right ilium. Very faint uptake in the 2^nd^ rib and 3^rd^ rib right antero-laterally, without any substrate on the diagnostic CT.

- Visceral Disease: Focal, PSMA expression in a spiculated lung nodule in the left upper lobe (15 mm), with pleural retractions.

**Conclusion**

1. Evidence of prostate cancer with intense PSMA expression in the peripheral dorsal zone of the right lobe, with suspicion of right seminal vesical invasion. Evidence of lymph node metastases in two right external iliac lymph nodes with intense PSMA expression. Evidence of bone metastasis in the left ilium, with intense PSMA expression in a sclerotic lesion on CT. Final miTNM: miT3bN1M1b.

2. Probably benign lesions 2nd and 3rd ribs left chest, with faint PSMA uptake (trauma?)

| **Anatomical location** | **mi TNM** | **Size** | **Number** | **PSMA**  **Expression Q** | **PSMA**  **Expression V** | **Reader Confidence** |
| --- | --- | --- | --- | --- | --- | --- |
| Right lobe + right seminal vesical | T3b | n/a | 1 | SUVmax 11 | Score 3 | 5 |
| External Iliac | N1 | 8 mm | 2 | SUVmax 5 | Score 3 | 5 |
| Right ilium | M1b | 14 mm | 1 | SUVmax 7 | Score 2 | 5 |
| 2^nd^ and 3^rd^ right ribs | n/a | n/a | 2 | SUVmax 2.1 | Score 1 | 2 |

3. High suspicion of a secondary (lung) malignancy in a spiculated 15 mm lung nodule in the left upper lobe. For adequate staging is an 2-[^18^F]FDG PET/diagnostic CT scan indicated.

| **Other Findings** | **Site** | **Size** | **Number** | **PSMA**  **Expression Q** | **PSMA**  **Expression V** | **Interpretation** |
| --- | --- | --- | --- | --- | --- | --- |
| Yes | Lung | 15 mm | 1 | SUVmax 4 | Score 1 | Primary lung malignancy |

Patient-2 (Name, Surname, Age, Date of birth)

**Patient History**

Prostate Cancer treated with robotic assisted radical prostatectomy (RALP) + pelvic lymph node dissection in 2/2020 Gleason Score 4+4, ISUP 4, pT3aN0Mx, R0, PNI+, initial PSA level 7.5 ng/mL. PSA nadir at 6 weeks detectable (0.37 ng/mL). PSA 4/2020 0.51 ng/mL. No on-going hormonal therapy. No salvage radiotherapy performed.

Anamnestic: none.

PSMA PET indication: biochemical persistence after radical prostatectomy.

**Technical information**

The patient was given 155 MBq [^68^Ga]Ga-PSMA-11 intravenously. Imaging of the body from the vertex to mid-thigh was obtained after 70 minutes with concurrent non-diagnostic CT for attenuation correction and anatomical correlation. Image acquisition was completed with late pelvic scan at 90 min p.i.

| **Radiotracer** | **Activity Injected** | **Uptake time** | **Type of Acquisition** | **CT Protocol** | **Contrast Oral/ IV** | **Diuretic** |
| --- | --- | --- | --- | --- | --- | --- |
| **[^68^Ga]Ga-PSMA-11** | 2.1 MBq/Kg | 70 min | Standard | Non Diagnostic | No | Yes  (10 mg) |

**Reporting of Findings**

This report has been produced in according to the European Association of Nuclear Medicine (EANM) standardized reporting guidelines v1.0 for PSMA PET/CT.

The physiological biodistribution of the radiotracer was regular.

- Prostate bed: no abnormal PSMA uptake detected.

- Lymph nodes: focal PSMA uptake in one presacral, right paramedian, lymph node (11 mm) and in one left pararectal lymph node (9 mm).

- Osseous disease: No focal positive PSMA lesions identified in the axial or appendicular skeleton.

- Visceral Disease: No focal positive PSMA lesions identified.

**Conclusion**

| **Anatomical location** | **mi TNM** | **Size** | **Number** | **PSMA**  **Expression Q** | **PSMA**  **Expression V** | **Reader Confidence** |
| --- | --- | --- | --- | --- | --- | --- |
| Presacral + Pararectal | N1 | 11mm  9mm | 2 | SUVmax 16 | Score 3 | 5 |

1. Evidence of lymph node metastases in two pelvic lymph nodes (presacral and pararectal) with intense PSMA expression. No further potential metastatic prostate cancer locations, expressing PSMA, have been detected.

Patient-3 (Name, Surname, Age, Date of birth)

**Patient History**

Prostate Cancer treated with robotic assisted radical prostatectomy (RALP) + pelvic lymph node dissection in 5/2014 Gleason Score 4+4, ISUP 4, pT3bN1Mx, R1, PNI+, initial PSA level 12.5 ng/mL. Adjuvant radiotherapy administrated (68Gy) in prostate bed + LH-RH agonists (6 months). PSA nadir <0.001 ng/mL. Biochemical recurrence in 6/2018 PSA 0.23 ng/mL rapidly increased up to 0.64 ng/mL 9/2018. Referred to PSMA PET/CT: positive in one external lymph node. PSMA guided SBRT performed. PSA undetectable. Further biochemical recurrence in 1/2020 (actual PSA 0.73 ng/mL; PSAdt 4.3 months). No ongoing hormonal therapy.

PSMA PET indication: biochemical recurrence after prostatectomy, SRT and SBRT.

**Technical information**

The patient was given 320 MBq [^18^F]PSMA-1007 intravenously. Imaging of the body from the vertex to mid-thigh was obtained after 90 minutes with concurrent diagnostic CT for attenuation correction and anatomical correlation.

| **Radiotracer** | **Activity Injected** | **Uptake**  **time** | **Type of Acquisition** | **CT Protocol** | **Contrast Oral/ IV** | **Diuretic** |
| --- | --- | --- | --- | --- | --- | --- |
| **[^18^F]PSMA-1007** | 4 MBq/Kg | 90 min | Standard | Diagnostic | No | No |

**Reporting of Findings**

This report has been produced in according to the European Association of Nuclear Medicine (EANM) standardized reporting guidelines v1.0 for PSMA PET/CT.

The physiological biodistribution of the radiotracer was regular.

- Prostate bed: no abnormal PSMA uptake detected.

- Lymph nodes: There are no PSMA avid, enlarged or abnormal nodes in the pelvis. Focal PSMA uptake in one lymph node in para-aortic region (14 mm).

- Osseous disease: Focal and intense PSMA uptake in sacrum (osteoblastic lesion 12 mm in CT). Further PSMA faint uptake in right femur without significant alterations in diagnostic CT.

- Visceral Disease: No focal positive PSMA lesions identified.

**Conclusion**

| **Anatomical location** | **mi TNM** | **Size** | **Number** | **PSMA**  **Expression Q** | **PSMA**  **Expression V** | **Reader Confidence** |
| --- | --- | --- | --- | --- | --- | --- |
| Para-aortic | M1a | 14 mm | 1 | SUVmax 8.6 | Score 2 | 5 |
| Sacrum | M1b | 12 mm | 1 | SUVmax 18.1 | Score 3 | 5 |
| Right Femur | M1b | n/a | 1 | SUVmax 7.1 | Score 2 | 4 |

Evidence of extra pelvic (para-aortic) lymph node and skeletal (sacrum) metastases with high PSMA expression. The further bone lesion in right femur, even if without CT alteration, is highly suspected for prostate cancer location (high PSMA expression).

1. **Synoptic Tables Template**

**Synoptic Table 1**

| **Radiotracer** | **Injected Activity** | **Uptake time** | **Type of Acquisition** | **CT Protocol** | **Contrast Oral/ IV** | **Diuretic** |
| --- | --- | --- | --- | --- | --- | --- |
|  |  |  |  |  |  |  |

**Synoptic Table – 2**

| **Anatomical location** | **mi TNM** | **Size** | **Number** | **PSMA**  **Expression Q** | **PSMA**  **Expression V** | **Reader Confidence** |
| --- | --- | --- | --- | --- | --- | --- |
|  |  |  |  |  |  |  |
|  |  |  |  |  |  |  |
|  |  |  |  |  |  |  |
